# Strategies to correct vaccine misinformation on social media for pregnant women and the impact of vaccine skepticism

**DOI:** 10.1038/s41598-025-98709-2

**Published:** 2025-04-30

**Authors:** Yi-Lun Jheng, Sander Van de Cruys, Larissa De Brabandere, Kirsten Maertens, Karolien Poels

**Affiliations:** 1 Department of Training and Educational Sciences, University of Antwerp, Antwerp, Belgium; 2 Department of Communication Studies, University of Antwerp, Antwerp, Belgium; 3 Antwerp Social Lab, University of Antwerp, Antwerp, Belgium; 4 Centre for the Evaluation of Vaccination, University of Antwerp, Wilrijk, Belgium

**Keywords:** Psychology, Human behaviour

## Abstract

Health-related misinformation on social media may affect vaccination behavior, particularly among (soon-to-be) mothers. Research suggested different strategies to correct misinformation, but it is unclear which strategies work best for what group and in what situation. Addressing the call for more emotion-based debunking strategies, this study examined how text genre (narrative vs. expository) and harm presence (with vs. without harm-stressing messages) interact to affect emotional responses, and debunking efficacy in corrective texts about vaccination and reproductive health, specifically targeting pregnant or planning-to-be pregnant women (*N* = 432) with varying levels of vaccine skepticism. We further assessed social media engagement, and information-seeking intentions. In particular, harm presence was tested as a moderator in the relationship between text genre and emotional responses, which in turn, mediate outcomes such as engagement with corrective texts and further information-seeking intentions on social media. Results from an online experimental survey showed that, in general, corrective texts about COVID-19 vaccine misinformation were more effective in reducing misconceptions compared to control texts. For women not skeptical towards vaccination, narratives with harm-stressing messages (vs. no harm) induced most negative emotions, reducing debunking efficacy, social media engagement, and information-seeking intention. For women skeptical towards vaccination, narratives (vs. expository) elicited stronger negative emotions, irrespective of harm-stressing messages, leading to decreased debunking efficacy, social media engagement, and the intention to seek information. This study illuminates the importance of tailoring vaccination corrective texts for different vaccine skepticism groups, avoiding one-size-fits-all strategies and being mindful of strong negative emotions and their counter-persuasive impact.

## Introduction

The World Economic Forum and the European Commission have identified massive online misinformation as one of the most pressing challenges for modern society^1,2^. What is at stake is a shared understanding of our reality, a precondition for democracy and conviviality in a society that through technological advancement has become both more globalized and more epistemically fragmented. Despite the wide availability of reliable and accessible scientific information, misinformation and radicalized beliefs thrive. Although the precise extent of the harmful role played by social media is still subject of intense debate^3,4^, scholars argue that, given the potentially destructive impact of misinformation on society and the speed with which online technology develops, the correlational evidence is sufficient to take urgent action now to mitigate the negative impact that social media might have on society^4^. This has become specifically apparent with regard to health-related misinformation in the context of COVID-19, in which social media have been described as “risk-attenuation and misinformation amplification stations”, where people were exposed to COVID-19 related misinformation making them less aware of the severity of the virus^5^. Indeed, the pandemic has shown that people invoke misinformation to support risky behavior (refusing to wear masks or to get vaccinations) that may endanger their own or other people’s health^6^. Furthermore, debunking science-related misinformation presents challenges, with correction effects being impacted by individuals’ familiarity with both sides of the issue and how politically polarized the issue is^7^. This paper thus concentrates on communication strategies aimed at fighting health-related misinformation within a social media context. Specifically, we test two strategies rooted in the field of health communication: narratives and harm-stressing messages. These strategies have been associated with eliciting emotional responses in health communication^8,9^.

Despite the fact that expecting mothers, fetuses, and newborns face an elevated risk of severe illness and mortality from various vaccine-preventable diseases, including COVID-19^10^, the majority of pre-marketing COVID-19 vaccine trials excluded pregnant participants. Given the unique vulnerabilities and informational needs of pregnant women and those planning to become pregnant, (soon-to-be) mothers emerge as a critical target group. Furthermore, it is indicated a high prevalence of perinatal anxiety, with the perinatal period demonstrating a surge in self-reported anxiety symptoms, escalating from 18.2% in the first trimester to 24.6% in the third trimester^11^. Pregnant women or new mothers may be vulnerable targets for false information about vaccination, given commonly felt uncertainties or anxieties at the prospect of giving birth to and caring for their child^12,13^. Vaccination-related misinformation indeed seems to be specifically targeted at women who plan to get (or already are) pregnant, by focusing on false claims about vaccines reducing fertility or leading to complications during or after pregnancy. The hesitant parents may also be easily swayed by false information they come across when deciding whether or not to vaccinate their child. Evidently, unvaccinated children are more likely to contract vaccine-preventable diseases and communities with a higher number of unvaccinated children are at risk of new outbreaks of those diseases (e.g., as seen for measles or pertussis^14^). Given that women’s reasons for refusing or doubting vaccinations are often based in legitimate concerns about the usefulness and safety of vaccination or in (lack of) confidence in the pharmaceutical or medical world^15^, it is important to guide them in the decision-making process with honest, accessible, actionable, and balanced information. However, no less important is the communication format and style of these messages (cf. a review of communication strategies to promote vaccination^16^). For example, health communicators should avoid talking down to or patronizing people when refuting vaccination misinformation on social media, especially because vaccine-skeptical mothers tend to be well-educated and not anti-science or unthinking in general^15,17–19^.

The psychology of misinformation has primarily focused on cognitive factors, but the roles played by emotion should not be underestimated^20,21^. Research has found that people who rely on emotions in their judgment are more likely to believe misinformation^22^. In light of the fact that misinformation often uses emotionally arousing content to provoke negative feelings and stoke online interactions^23,^^24^, exploring emotion-based strategies for debunking misinformation is essential. When it comes to combating misinformation, corrective texts have been investigated for their tone. For instance, a study investigated the impact of adopting varying tones in corrections, such as using derogatory language towards the original misinformation in a correction (uncivil tone) or expressing that participants’ potential concerns are understandable, while gently asserting that the presented information is incorrect (affirmative correction)^25^. Another study evaluated corrective texts with humor vs. without humor and found that people perceived the non-humor corrective message to be more credible, which resulted in fewer HPV misperceptions^26^. Although message tone has been investigated, the efficacy of different genres of text for corrective texts is still unclear. Text genre has been found to be important in health communication^27,28^. For example, vaccination messages conveyed by mothers’ narratives may be effective in persuading mothers to boost HPV vaccination, since stories told by fellow mothers may resonate with them^29^. Yet, the role text genre plays in debunking misinformation is largely unexplored. A prior study focusing on e-cigarette misinformation utilized text genre for correction purposes and found that narrative content induced higher emotional involvement compared to non-narrative content, but did not reduce resistance to corrective messages^30^. Furthermore, although the widespread use of fear appeals in health communication, the interaction between text genre and the inclusion of harm-stressing messages remains relatively unexplored. Harm-stressing messages that emphasize harmful consequences can increase awareness of health risks^31^. However, when included in narratives, these messages might exacerbate perceived harm because people can better relate to the suffering, potentially causing a backlash. Moreover, vaccine skepticism is an attitude or belief that might result in behaviors such as delaying or refusing recommended vaccines^32^. Nevertheless, it remains unknown how different corrective texts are processed differently by vaccine skeptics and non-skeptics.

The present study aimed to examine the effectiveness of corrective texts, employing different text genres (narrative vs. expository) and varying harm levels (with vs. without harm-stressing messages), for reducing vaccination-related misconceptions and engaging women of childbearing age with corrective information on social media. Furthermore, we investigated the differential impact of corrective texts among vaccine-skeptical and non-skeptical groups of (soon-to-be) mothers.

RQ1: To what extent do corrective texts demonstrate greater debunking efficacy than control texts?

RQ2: (1) Do emotional responses mediate the relationship between text genre and outcomes (debunking efficacy, social media engagement, information seeking intention)? (2) How do harm-stressing messages moderate the relationships between text genre, emotional responses, and these outcomes, and 3) How do these effects differ between vaccine skeptics and non-skeptics?

### Misinformation, debunking, and corrective texts

The growing recognition of the detrimental effects of misinformation, particularly in the wake of the COVID-19 pandemic, has spurred an increased focus on strategies to address misconceptions. Among these, debunking has become as a key approach^33^. The underlying process of the debunking approach (i.e., presenting misinformation followed by corrective messages, as applied in this study) is explained by the Knowledge Revision Components (KReC) framework^34^. This framework posits that newly encoded correct information gradually weakens misconceptions within the integrated network, lessening the influence of previously acquired misinformation^34^. However, the weighting process between pre-existing beliefs and incoming information may be affected also by various factors, such as the psychological characteristics of the receiver and the perceived ability of the source^35^.

Debunking efficacy refers to the effectiveness of corrective efforts in reducing misinformation endorsement. It is operationalized as the difference between belief after exposure to corrective information and initial belief in misinformation. A recent meta-analysis supports the efficacy of debunking refutational messages over non-refutational ones in experimental settings addressing scientific misconceptions. However, no significant moderating effects (e.g., individual or setting characteristics) were observed on the effectiveness of refutation messages^36^. In the domain of health communication, empirical studies have also shown that debunking strategies (e.g., visual aids from various sources) are effective in reducing misconceptions or diminishing belief in misinformation^37,38^. Building on the theoretical foundations of the KReC model and the existing body of research, we propose the following hypothesis:

Hypothesis (H1): The debunking effect of corrective texts is more effective than that of control texts.

### Emotional responses, text genre, and the moderating role of harm presence

Emotions are integral to information processing and shaping behavioral intentions. Inspired by the Elaboration Likelihood Model (ELM)^39^, the Cognitive Reconstruction of Knowledge Model (CRKM)^40^integrates the “hot” construct into the conceptual change process. Specifically, both the message and recipient characteristics (e.g., motivation and affect) influence cognitive engagement, thus driving conceptual change^40^. For example, when corrective messages contradict personal identity, they can evoke negative emotions and a sense of threat, reinforcing resistance to corrective information and hindering the revision of misconceptions^41^. Prior research has highlighted that emotions may act as a mediator between text genre and behavioral intention^42,43^. Research in health communication suggests that narratives are more effective in increasing knowledge and attitudes than dry, didactic texts when conveying health-related information^8,44,45^. While text genres have been studied for their persuasive effects when providing self-contained information, little is known about their effectiveness in counteracting misinformation. A possible reason narrative texts may be more persuasive is that they elicit higher emotional engagement^30^. People tend to rely more on anecdotal evidence especially in situations that are health-related, personal, or potentially serious^46^. Using narratives may thus help readers to envision concretely (and so better grasp) the corrective information of COVID-19 vaccination. Another reason may be that narrative texts are also perceived as less directive because they only contain anecdotal information without didactically constructed arguments, making them appear less overtly persuasive (less manipulative) or less authoritative compared to expert-written, fact-based texts. Indeed, narratives were perceived as less threatening to readers’ autonomy (compared to non-narratives)^47^. It was found that autonomy-supportive language, compared to imperative language, prompted lower levels of anger and more favorable decisions regarding flu vaccination^48^. Relatedly, a meta-analysis on debunking showed that detailed texts (vs. texts merely stating the misinformation is false) may also induce higher “misinformation persistence”, meaning that false beliefs persist after the misinformation has been rebutted^49^. Such persistence effects (or even backfire effects: increased misbelief after debunking) suggest that more arguments are not necessarily better (cf. “overkill backfire”^20^).

A substantial body of research has examined the relative effectiveness of narratives and expository texts, yet findings sometimes show inconsistent^8,50^. A meta-analysis has highlighted the need for further investigation into potential moderating variables that may account for these discrepancies^51^. In this regard, research stresses the importance of message features as potential moderators of narrative effects^52^. For example, empirical evidence indicated that message framing (i.e., emphasizing the positive consequences of quitting an unhealthy behavior vs. the negative consequences of maintaining it) moderates the influence of text genre on risk perception. Specifically, narratives that emphasize the negative consequences of continuing an unhealthy behavior has found to elicit greater perceived severity^53^. This finding implies that messages stressing negative consequences strengthen the relationship between narrative communication and risk perception. Given the widespread use of fear-evoking campaigns and threat-based messages in health communication, it is essential to understand their moderating role in narrative persuasion, particularly within debunking contexts. On the one hand, embedding messages that emphasize harmful consequences in narratives may enhance emotional engagement (increasing empathy and risk perception)^53^, so such messages could be more effective in neutralizing misinformation. For instance, refutation texts augmented with negative emotional content seem to stimulate the cognitive processes involved in the knowledge revision, whereas refutation texts with positive emotional content seem to dilute attention and thus weaken the integration of corrective information in debunking misinformation^54^. There may also be an interaction of harm focus with text genre, for example because it is more natural to focus on personal suffering in a narrative text. In addition, in contentious (political) debates, research has found that it is easier to find common ground when focusing on personal experience and (avoidable) harm than when merely focusing on facts and statistics^55^.

While the inclusion of harm-stressing messages in corrective texts may strengthen the relationship between text genre and emotional response, they might also be perceived as deliberately playing on emotions, potentially undermining debunking efficacy. Prior research suggests that people are aware of the persuasive intent of corrective messages, especially when presented after the misinformation, the effectiveness may be impaired^30^. A similar pattern can also be observed in text genre where narratives lose their advantage over expository messages when manipulative intent is salient^56^. Additionally, fear appeals hinge on the idea that confronting people emotionally with the negative consequences of their actions will drive behavioral change, but it’s not a panacea and only works in specific circumstances^57^. The communication during the COVID-19 pandemic has precisely been criticized for using fear appeals because people often were already stressed and anxious in this unprecedented, global emergency state^58^. On top of this, (soon-to-be) mothers, who commonly experience heightened levels of perinatal anxiety, might be particularly sensitive to harm-stressing messages.

Hypothesis (H2): Emotions mediate the relationship between text genre and outcomes (debunking efficacy, social media engagement, and information-seeking intentions).

Hypothesis (H3): Harm-stressing messages moderate the effect of text genre on emotions, thereby strengthening or weakening the indirect effects of text genre on outcomes (moderated mediation).

### Vaccine skepticism and its impact on debunking efficacy, social media engagement, and information seeking intention

Vaccine skepticism, often fueled by misinformation, poses a significant challenge for public health efforts by reducing vaccination acceptance^59,60^. The decline in vaccination uptake rates has been linked to the spread of misinformation online^61^. Social media plays a crucial role in the spread and correction of misinformation, as engagement behaviors—such as liking and sharing—reflect how individuals interact with and endorse (mis)information online^62,63^. However, the emotional responses individuals have to a message can influence how they engage with it. Research has shown that negative emotions (e.g., fear and anxiety) and psychosocial stress are associated with an increased intention to avoid information^64,65^, which may further discourage individuals from seeking accurate information instead of fostering engagement. According to the valence approach of affect on judgment, messages that elicit negative emotions can result in negative judgments^66^. When a corrective message is perceived as too threatening and feel unable to respond effectively, individuals often engage in fear control processes, where they are motivated to avoid fear by using defensive avoidance^67^. For non-vaccine skeptical (soon-to-be) mothers, the combination of the COVID-19 threat and the stress of pregnancy can be overwhelming, not only that, harm-stressing messages would exacerbate negative emotions, causing them to focus on their fear and resort to defensive avoidance to cope. These emotional responses can lead them to focus on their fears rather than engage with and seek further corrective health information. Moreover, vaccine skepticism is driven by broader psychological and cultural values, making skeptics view pro-vaccine messaging as arrogant and motivated by vested interests rather than grounded in evidence^32^. Also, conflicting corrections that challenge individuals’ identities can evoke negative emotions, hindering knowledge revision^41^. For vaccine skeptics, messages that contradict their existing beliefs may provoke negative emotions and reactance. This reactance can prompt them ignore or be reluctant to seek further information that might challenge their views.

Cognitive and socio-affective factors, such as intuitive thinking, emotional states, and worldview, may affect individuals’ susceptibility to misinformation^68^. Efforts to counter misinformation through corrective messages may be influenced by cognitive and emotional factors. For example, intrinsic barriers to COVID-19 information, as measured by a literacy-sensitive scale, capture the cognitive and affective barriers challenges individuals have faced in past health information searches related to COVID-19, including effort, frustration, concerns about information quality, and comprehension difficulties^69^. Such barriers may lead to information avoidance, reducing further engagement with corrective messages. This issue may be particularly relevant for pregnant women who are often driven by concerns over vaccine safety and potential risks to the fetus^70^. Relatedly, research suggests that women who have experienced pregnancy complications tend to engage in more active health information-seeking behavior, potentially as a strategy to mitigate risks^71^. To account for these influences, our study controls for two key variables: intrinsic barriers to vaccine information and pregnancy complications, offering a clearer understanding of how individuals with varying vaccine skepticism engage with different types of corrective messages.

*Hypothesis (H4): A corrective text inducing negative emotions decreases debunking efficacy, social media engagement, and information-seeking intentions, with harm-stressing messages particularly triggering stronger negative emotions in vaccine non-skeptics.*

## Results

### The basic debunking efficacy of corrective (vs. non-corrective control) texts

**Fig. 1:**
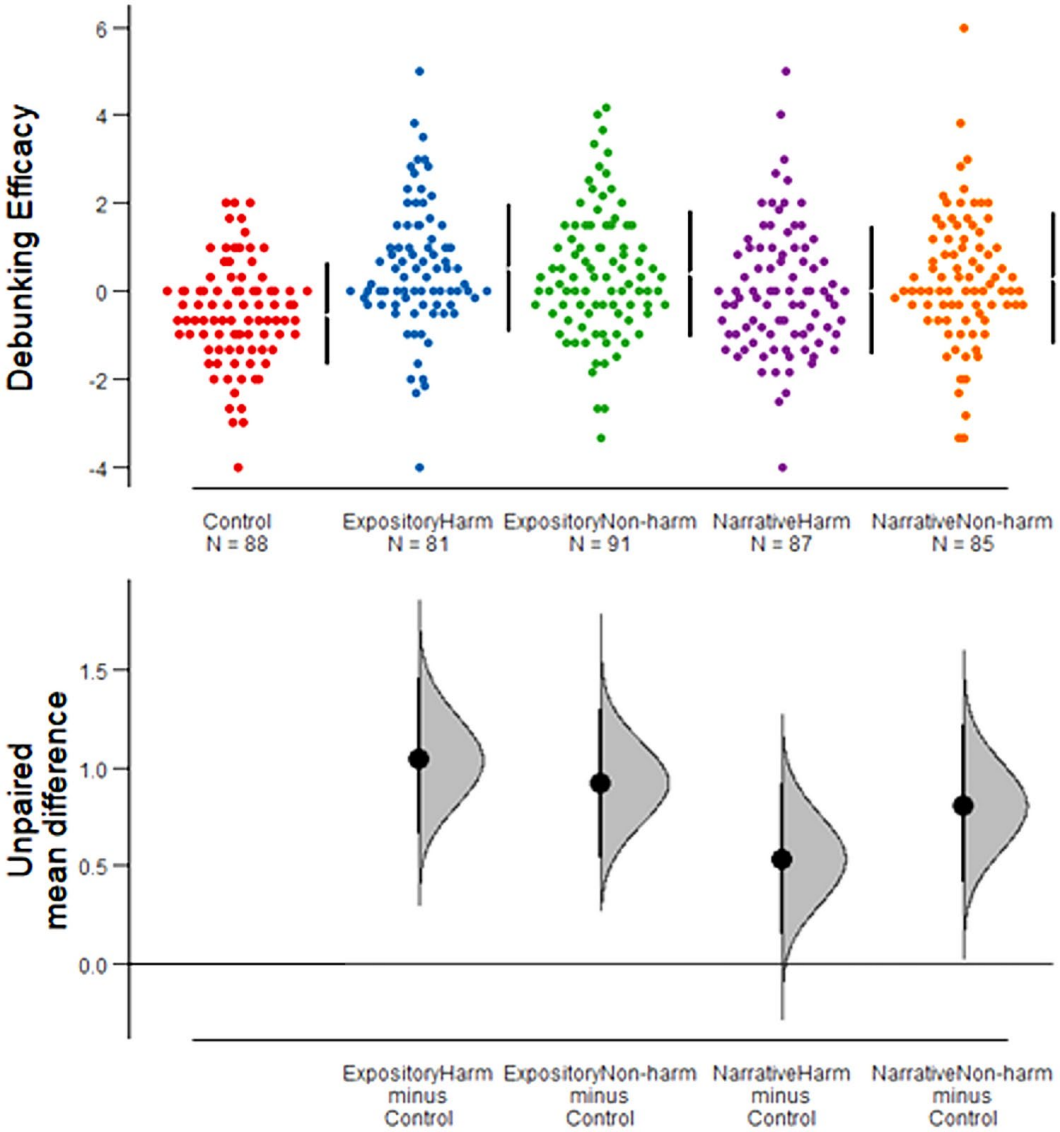
The estimation graphic shows the comparison of mean differences in debunking efficacy between corrective texts and ***non-***corrective control text condition. The 95% confidence interval of mean differences is illustrated.

A One-Way Analysis of Variance (ANOVA) was performed to assess debunking efficacy across all participants (Appendix Table 4 and Table 5). The results revealed a significant difference in debunking efficacy among the text conditions with a medium effect size, *F*(4, 427) = 7.55, *p* < 0.001, *η*^*2*^ = 0.07. Figure 1 shows the mean difference in debunking efficacy between each corrective condition and the non-corrective control condition. Results indicated that the only condition that did not perform better than a non-corrective text was the narrative with harm corrective text (NH) (*M* = -0.00, *SD* = 1.45). Generally, debunking works but not for the NH. The debunking efficacy was significantly higher for participants exposed to expository with harm corrective text (EH) (*M* = 0.51, *SD* = 1.46), expository without harm corrective text (EnoH) (*M* = 0.38, *SD* = 1.44), and narrative without harm corrective text (NnoH) (*M* = 0.27, *SD* = 1.48) than that for those exposed to non-corrective control condition (*M* = -0.53, *SD* = 1.17).

### Three-way interaction

**Fig. 2:**
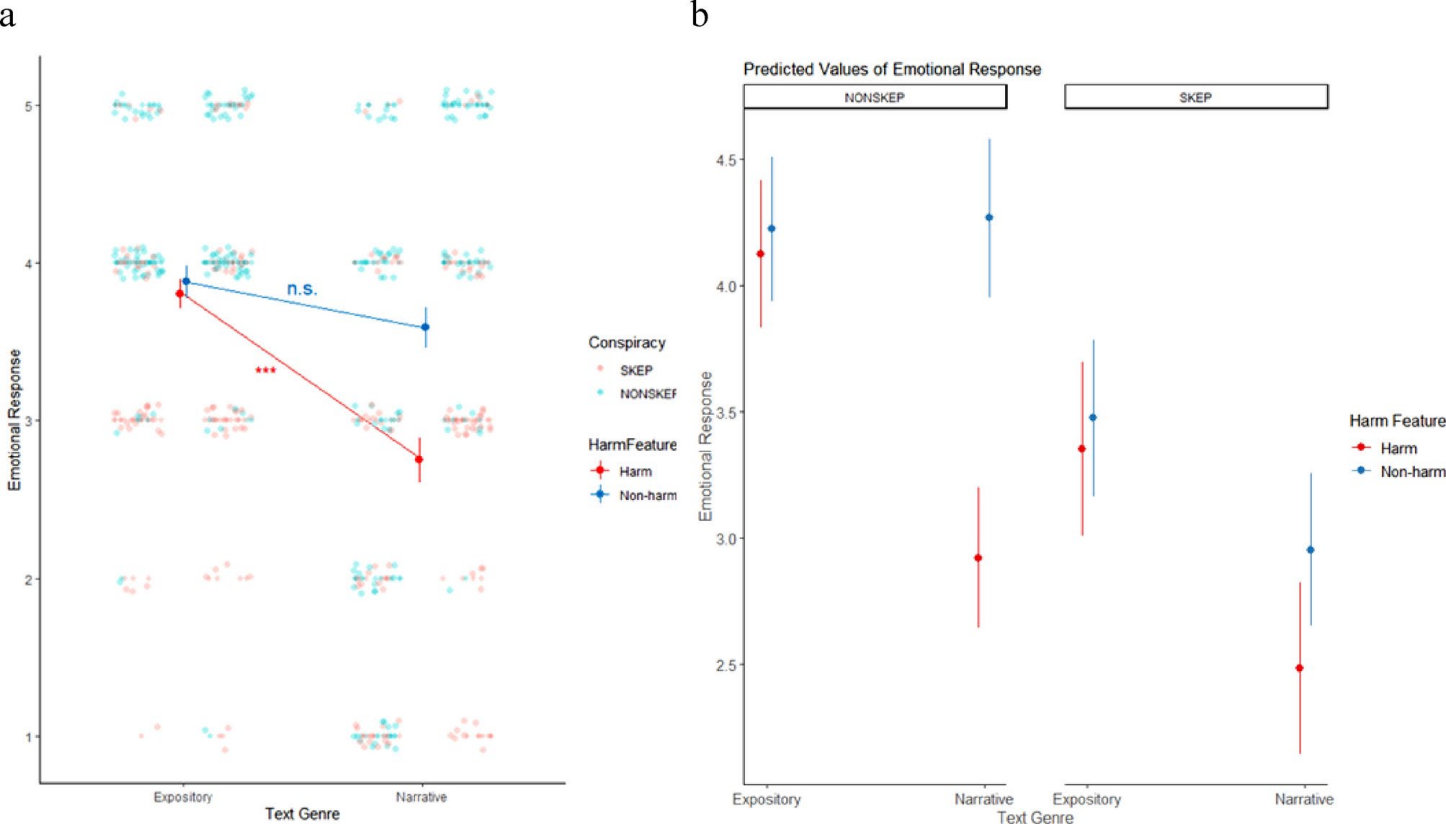
Interaction effect of *text genre* and *harm presence* (with harm-stressing messages: read line; without harm-stressing messages: blue line) on emotional responses, along with the emotional responses based on *vaccine skepticism* (SKEP: pink points; NONSKEP: green points).

To examine the interplay between recipient characteristics (vaccine skepticism) and message characteristics (genre and harm presence), we first performed a three-way interaction analysis to assess their collective impact on emotional responses. R version 4.1.2 was used to conduct the Aligned Rank Transform (ART) ANOVA analysis because it allows the handling of data that is not normally distributed and homoscedasticity is violated^72^. Table 1 reveals that there is a statistically-significant difference in emotional responses by text genre (*F*(1) = 35.99, *p* < 0.001, *ηp*^*2*^ = 0.10), by harm presence (*F*(1) = 26.24, *p* < 0.001, *ηp*^*2*^ = 0.07), and by vaccine skepticism (*F*(1) = 47.54, *p* < 0.001, *ηp*^*2*^ = 0.12). Additionally, the interaction effect of text genre and harm presence on emotional responses was found to be significant (*F*(1) = 9.81, *p* = 0.002, *ηp*^*2*^ = 0.03), whereas neither the interaction effect of text genre and vaccine skepticism nor the interaction effect of harm presence and vaccine skepticism on emotional responses was significant (*F*(1) = 0.38, *p* = 0.54, *ηp*^*2*^ = 0.001; *F*(1) = 0.54, *p* = 0.46, *ηp*^*2*^ = 0.002). Figure 2a illustrates that corrective texts containing harm-stressing messages led to a more negative emotional response when formatted in a narrative style (*M* = 2.75, *SD* = 1.34) compared to in an expository style (*M* = 3.80, *SD* = 0.84). Moreover, the model shows a statistically significant three-way interaction effect among text genre, harm presence, and vaccine skepticism (*F*(1) = 5.53, *p* = 0.02, *ηp*^*2*^ = 0.02) on emotional response. To enhance the understanding of the trilateral interplay, Fig. 2b delineates the interplay among text genre, harm presence, and vaccine skepticism. Among skeptical individuals, no significant distinction in emotional responses was observed between texts containing harm-stressing messages and those without such messages when presented in a narrative format. In contrast, non-skeptical individuals displayed statistically significant greater negative emotional responses when exposed to narrative texts incorporating harm-stressing messages as opposed to their non-harm versions.

**Table 1:** Three-way interaction model on emotional responses.

Source of variation	Df	Sum of squares (SS)	Residual sum of squares (RSS)	F value	*Pr(*> *F)*
Text Genre	1	324,011.30	3,025,347.00	35.99	** < 0.001**
Harm Presence	1	243,162.90	3,113,804.00	26.24	** < 0.001**
Vaccine Skepticism	1	410,968.60	2,904,375.00	47.54	** < 0.001**
Text Genre x Harm Presence	1	95,336.60	3,264,245.00	9.81	**0.002**
Text Genre x Vaccine Skepticism	1	3785.80	3,342,026.00	0.38	0.54
Harm Presence x Vaccine Skepticism	1	5308.00	3,284,367.00	0.54	0.46
Text Genre x Harm Presence x Vaccine Skepticism	1	53,443.30	3,247,176.00	5.53	**0.02**

Singnificant values are in bold.

### Moderated mediation model for NONSKEP

**Fig. 3:**
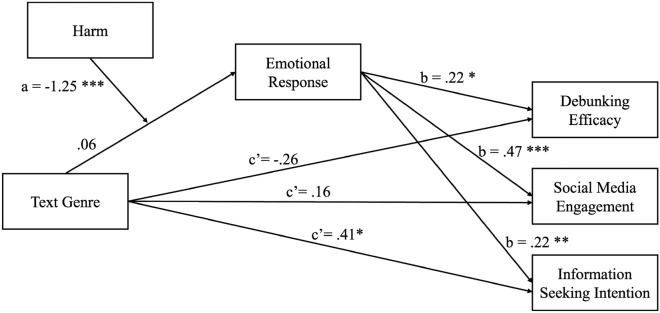
Multiple moderated mediation analysis for NONSKEP. *p < 0.05; **p < 0.01; ***p < 0.001.

Results of the hypothesized moderated mediation models on *debunking efficacy, social media engagement, and information seeking intention*using PROCESS macro model 7^73^ are presented in Table 2 and Fig. 3. Regarding *debunking efficacy*, for the a-path from text genre to emotional responses, there was a significant interaction between text genre and harm presence (*b* = -1.25, *p* < 0.001, *ΔR*^*2*^ = 0.08) (Table 2). The conditional effect of text genre on emotional responses was significant for conditions with harm (*b* = -1.20, *p* < 0.001), but it was not significant for conditions without harm-stressing messages (*b* = 0.06, *p* = 0.78). The b-path from emotional responses to debunking efficacy was significant (*b* = 0.22, *p* = 0.02), revealing greater positive emotional responses were associated with higher debunking efficacy. The overall moderated mediation model was supported (*b* = -0.28, 95% CI [-0.55, -0.05]), indicating the indirect effect via emotional responses was significantly moderated by harm presence in genres of texts. The (negative) conditional indirect effect was significant for the text conditions with harm-stressing messages, *b* = -0.26, 95% CI [-0.51, -0.05], but not for those without harm-stressing messages, *b* = 0.01, 95% CI [-0.06, 0.10].

**Table 2:** Moderation mediation analysis for NONSKEP.

Variable	Model a-path			Debunking efficacy			Social media engagement			Information seeking intention		
				Model b/c’-path			Model b/c’-path			Model b/c’-path		
	*β*	*SE*	*p*	*β*	*SE*	*P*	*β*	*SE*	*P*	*β*	*SE*	*P*
Text genre	0.06	0.20	0.78	− 0.26	0.20	0.20	0.16	0.15	0.29	0.41	0.17	**0.02**
Harm presence	− 0.09	0.20	0.67									
Text genre x Harm presence	− 1.25	0.28	** < 0.001**									
Emotional response				0.22	0.09	**0.02**	0.47	0.07	** < 0.001**	0.22	0.08	**0.004**
Pregnancy complications (control)	0.13	0.19	0.48	0.43	0.26	0.10	0.10	0.19	0.60	0.08	0.22	0.73
Intrinsic barriers to COVID-19 information (control)	0.06	0.08	0.49	0.02	0.11	0.86	0.17	0.08	**0.04**	0.31	0.10	**0.002**

Significant values are in bold.

In terms of *social media engagement*, the effect of emotional responses on social media engagement was significant (b-path), showing that higher positive emotional responses were correlated with increased social media engagement (*b* = 0.47, *p* < 0.001). The overall moderated mediation model was supported (*b* = -0.59, 95% CI [-0.92, -0.30]). The conditional indirect effect of text genre on social media engagement via emotional responses was found for those in text conditions with harm-stressing messages (*b* = -0.56, 95% CI [-0.84, -0.33]), but not for those without harm-stressing messages (*b* = 0.03, 95% CI [-0.12, 0.19]).

As for *information seeking intention*, the b-path from emotional responses to information seeking intention was significant (*b* = 0.22, *p* = 0.004), revealing greater positive emotional responses were associated with higher intention to seek for information. The moderated mediation model was supported overall based on the index of moderated mediation (*b* = -0.28, 95% CI [-0.58, -0.05]). The indirect effect of text genre on information seeking intention through emotional responses was significant when corrective texts contained harm-stressing messages (*b* = -0.27, 95% CI [-0.52, -0.05]). However, when corrective texts did not include harm-stressing messages, this indirect effect was absent (*b* = 0.01, 95% CI [− 0.07, 0.11]).

### Moderated mediation model for SKEP

**Fig. 4:**
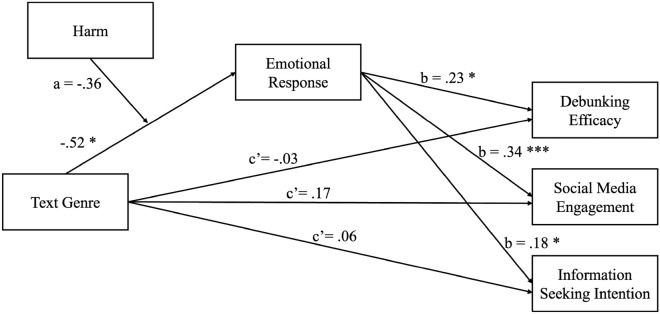
Multiple moderated mediation analysis for SKEP. *p < 0.05; **p < 0.01; ***p < 0.001.

The moderated mediation models on *debunking efficacy, social media engagement, and information seeking intention*among vaccine skeptics was also tested with PROCESS macro model 7^73^ (Table 3 and Fig. 4). Concerning *debunking efficacy,* a significant effect of text genre on emotional responses was observed (*b* = -0.52, *p* = 0.03), revealing fewer positive emotional responses toward narrative corrective texts (*M* = 0.26, *SD* = 1.64) compared to expository ones (*M* = 0.43, *SD* = 1.50). Additionally, there was a significant effect of emotional responses on debunking efficacy (*b* = 0.23, *p* < 0.05), indicating that higher levels of positive emotional responses were associated with increased debunking efficacy. However, the moderated mediation model was not overall supported (*b* = -0.08, 95% CI [-0.32, 0.09]), as the harm presence did not play a moderating role in the relationship between text genre and emotional responses. In terms of the *social media engagement,* there was a significant effect of text genre on emotional responses (*b* = -0.52, *p* = 0.03). Besides, greater positive emotional responses were associated with increased social media engagement (*b* = 0.34, *p* < 0.001). However, neither harm presence nor the interaction between text genre and harm presence showed statistically significant effects on emotional responses, the overall moderated mediation model was then not supported (*b* = -0.12, harm presence 95% CI [-0.39, 0.10]). Regarding *information seeking intention*, the effect of emotional responses to information seeking intention was significant (*b* = 0.18, *p* = 0.04), revealing greater positive emotional responses was associated with higher intention to seek for information. The overall moderated mediation model was not supported (*b* = -0.06, 95% CI [-0.25, 0.06]), as there was no significant moderating role of the harm presence on the relationship between text genre and emotional responses.

**Table 3:** Moderation mediation analysis for SKEP.

Variable	Model a-path			Debunking efficacy			Social media engagement			Information seeking intention		
				Model b/c’-path			Model b/c’-path			Model b/c’-path		
	*β*	*SE*	*p*	*β*	*SE*	*P*	*β*	*SE*	*P*	*β*	*SE*	*P*
Text genre	− 0.52	0.23	**0.03**	− 0.03	0.26	0.91	0.17	0.17	0.33	− 0.06	0.20	0.77
Harm presence	− 0.11	0.25	0.67									
Text genre x Harm presence	− 0.36	0.35	0.31									
Emotional response				0.23	0.12	** < 0.05**	0.34	0.08	** < 0.001**	0.18	0.09	**0.04**
Pregnancy complications (control)	− 0.13	0.24	0.58	− 0.47	0.34	0.17	-0.11	0.22	0.64	− 0.19	0.26	0.46
Intrinsic barriers to COVID-19 information (control)	0.01	0.12	0.94	0.13	0.17	0.45	0.18	0.11	0.10	0.46	0.13	** < 0.001**

Significant vlaues are in bold.

## Discussion

Misinformation circulates widely on social media nowadays. In light of this, we first examined the effectiveness of corrective messages in debunking misinformation on social media in the context of COVID-19 communication among a sample of (soon-to-be) mothers. Further, we investigated the impact of the interplay between text genres and harm presence within corrective texts on debunking misinformation, emotional responses, social media engagement, and information seeking intention. Ultimately, we gained insights into how vaccination skepticism among women of childbearing age affects the effectiveness of corrective texts.

### The significance of delivering corrective messages

Recent reviews suggest that debunking or correction of misinformation often works, although there is considerable discussion on the size and generality of its effects^20,36,49,68^. In this study, a medium effect size for the impact of corrective messages on debunking was observed, consistent with the effect sizes commonly seen in strategies addressing misinformation related to health and the environment, which typically demonstrate effect sizes ranging from small to medium^74^. This study found that corrective texts are generally more effective at debunking misinformation than unrelated control messages that did not provide any corrections. In line with previous studies, correction explanations help to reduce false beliefs (induce knowledge revision), while the format of the message seems to be less important^54,75,76^. In a study conducted by Swire-Thompson et al.^76^, the influence of various explanation formats on misinformation belief ratings was investigated. The different formats included variations in the order of claim and affirmation/retraction, as well as labeling of misinformation/facts as false/true in their claims with retractions/affirmations attached. The results indicated that participants exposed to all the explanation formats consistently reported significantly lower misinformation belief ratings compared to those in the control condition without any explanation, but different formats did not differ significantly. Similarly, Kotz et al.^75^, examined the influence of text structures (the “truth sandwich” approach, where the misinformation is placed in the middle block to mitigate the primacy and recency effects vs. “bottom-heavy” approach, which presents the misinformation at the outset followed by sections containing debunking information) on agreement with misinformation about COVID-19 vaccines. The findings revealed that the debunking messages effectively diminish belief in the targeted misinformation. However, there were no significant differences in text structures or formats regarding agreement with misinformation statements. Also, a study examined the impact of adding emotional content in refutation texts addressing misconceptions about vaccines. The results demonstrated that refutation texts, whether including positive, negative or lacking emotional content, worked (with no significant differences between refutation conditions), as compared to non-refutational control texts^54^.

### The interplay between text genre and harm presence in relation to outcome variables through emotional responses

As expected, different corrective messages can elicit negative emotions depending on the (soon-to-be) mothers’ vaccine skepticism. For the NONSKEP group, we found the effect of text genre on emotional responses to corrective texts depended on the presence of harm presence. Specifically, when narratives were coupled with harm-stressing messages, which emphasized adverse consequences of not getting vaccinated and being infected with COVID-19, individuals tended to experience significantly more negative emotions. Consequently, this heightened negativity decreased debunking efficacy, reduced the likelihood of individuals liking and sharing such posts on social media, and lowered their inclination to seek additional related information. An explanation could be that COVID-19 may have caused people to become overwhelmed with stress and anxiety. It was found that the psychological distress reported by pregnant women during the COVID-19 pandemic was notably higher than that reported by pregnant women before the pandemic^77^. Thus, the use of fear appeals in COVID-19 communication may produce unintended adverse consequences^58^. Study also showed posters with fear appeals exhibited lower levels of information believability and COVID-19 vaccination intention than those without fear appeals^78^. The process of reading narratives involves mental imagery^79^. The harm-stressing messages in narratives present distressing and unpleasant scenes (in our case, a woman succumbing to COVID-19 in the ICU) making them more vivid and unsettling for the audience. Moreover, the situation of pregnancy may already be a stressor. (Soon-to-be) pregnant women may be more attuned to harm-stressing messages, which may lead to unfavorable responses. Therefore, it may be best to refrain from using narratives with harm-stressing messages when tackling pregnancy or newborn-related health misinformation among non-vaccine skeptics.

Individuals adhering to anti-vaccination attitudes show a less negative response to statistical information alone than to anecdotal testimonies alone^80^. In line with that, this study found that for the SKEP group, narratives (vs. expository), regardless of whether they included a harm presence or not, elicited more negative emotional responses. These responses, in turn, lower debunking efficacy, social media engagement, and information-seeking intention. One possible reason for the observed reduced effectiveness of narrative corrective texts compared to expository texts could be related to the possibility that narratives might divert readers’ attention away from critical correcting information. This diversion becomes particularly impactful when the essential correction details are not seamlessly integrated into the central, attention-grabbing elements of the narrative. Consequently, distraction exercises a challenge in effectively delivering essential correction information within narrative formats. Moreover, the skeptics induced more unfavorable emotional reactions to narrative corrective texts and in turn showed a lower likelihood of endorsing the information by liking or sharing it on social media. An explanation may be that individuals who are skeptical about vaccines may perceive narrative corrective texts as intentionally playing on emotions, thus viewing them as more manipulative in nature. This finding aligns with literature on the affective resistance of narrative persuasion, where narratives evoked affective resistance, and manipulative intent were found to amplify participants’ affective resistance^81^. Women with vaccine skepticism may interpret narrative corrective information (with or without harm) as manipulative attempt, causing them to dismiss the information.

This study is not without limitations, particularly regarding internal and external threats to validity. One internal validity concern is selection bias, as pre-existing differences among participants (e.g., prior exposure to misinformation) could have influenced the results. Additionally, since this study only captures the immediate effects of the corrective messages, we cannot determine whether participants revert to prior beliefs over time. This limitation might impact internal validity, as the lack of long-term measurement makes it challenging to assess whether the effect is temporary or genuine. Future research could use longitudinal designs with follow-up measures to evaluate the lasting influence of corrective messages. Regarding the threats to the external validity, this study was conducted in a controlled environment using a simulated social media layout with artificial stimuli. While this allowed for the manipulation of specific variables, it may not fully capture real-world interactions. To enhance ecological validity, future research could utilize readily available real-world materials, such as popularly spread misinformation and corrections from authoritative institutions or news media. Moreover, conducting studies in more authentic settings, such as actual interactive social media platforms, could provide deeper insights into how people engage with corrective information in natural contexts. In addition, the current study targeted English-speaking (soon-to-be) mothers for delivering corrective messages. As a result, the sample may not be representative of other cultural or linguistic groups, which limits the generalizability of our intervention messages. Future research could replicate the experiment using the same corrective messages to assess whether the effectiveness of corrective interventions may differ across various cultures, languages, and socio-political contexts, thereby providing more robust insights into combating misinformation on a global scale. Another limitation of this study is its primary focus on COVID-19 vaccines, which may limit the generalizability of the findings to other vaccines. Given the distinct patterns of vaccine hesitancy observed across different vaccine types, it is crucial to acknowledge the varying perceptions of vaccines and their impact on public health campaigns. For example, parents tend to be more hesitant about the COVID-19 vaccine due to lower confidence in its effectiveness, safety, and necessity for their children compared to routine childhood vaccines^82^. Relatedly, research has shown that the effectiveness of vaccine communication varies depending on the type of vaccine and the context in which it is administered^83^.

This study examines how the types of corrective messages (“what”) are perceived and processed, and how this, in turn, influences debunking efficacy, social media engagement, and individuals’ intention to seek further information. While we consider vaccine skepticism an important personal factor, we acknowledge that various other elements may also affect how individuals interpret the information. People do not receive information in isolation. The way we respond to corrective messages and misinformation is shaped by several contextual factors, such as the perceived trustworthiness of the source, the credibility of the content, and individual differences^84^. To improve vaccine acceptance and preparedness for future outbreaks, researchers should closely investigate the factors contributing to varying levels of vaccine hesitancy across different vaccine types, along with the effectiveness of tailored communication messages in diverse contexts.

## Conclusion

This study examined how the inclusion or exclusion of harm-related elements within genres of corrective texts may affect debunking efficacy, social media engagement, and information-seeking intention in the context of communicating COVID-19 vaccination and reproductive health information to a sample of (soon-to-be) mothers. Amidst the COVID-19 pandemic, we found that there is little advantage to using narratives to get women to correct misbeliefs about vaccination. A straightforward, clear, and fact-based approach to counter misinformation without an excessive emotional charge seems the best bet. More importantly, the study sheds light on the subtle yet powerful influence of narrative and harm presence on distinct audience segments. While harm-stressing messages prove to be unfavorable among non-skeptical women, the narrative format emerges as a trigger for negative emotional responses in vaccine skeptics. These findings indicate that avoiding texts that provoke too strong negative emotional responses is crucial, as they may cause counter-persuasion. This study also suggests there is no one-size-fits-all approach to combating online misinformation. Health communicators and educators must be acutely aware of the impact negative emotions have on communication effectiveness and need to tailor corrective texts, attuned to the unique sensitivities of specific audiences.

## Methods

All experimental protocols were approved by the Ethics Committee for the Social Sciences and Humanities of the authors’ affiliation (Ethics ID: SHW_22_014). All methods were performed in accordance with the relevant guidelines and regulations. The experimental text materials, anonymous data and scripts can be found on the Open Science Foundation (OSF).

### Participants

We recruited 432 English-speaking pregnant or planning-to-be pregnant women (between 18–45 years of age; mean age = 27.77; *SD* = 5.88) through Prolific.co. To ensure a diverse representation of COVID-19 vaccine attitudes, we used Prolific’s “COVID-19 Vaccine Opinions” prescreening filter. Participants were categorized as *For* (positive attitude), *Against* (negative attitude), or *Neutral* (no strong opinion). Using this purposive sampling, we attempted to recruit equal numbers from the *For* group and the *Against/Neutral* group. All participants provided informed consent prior to participating and received £3.75 for their participation.

### Materials

**Fig. 5:**
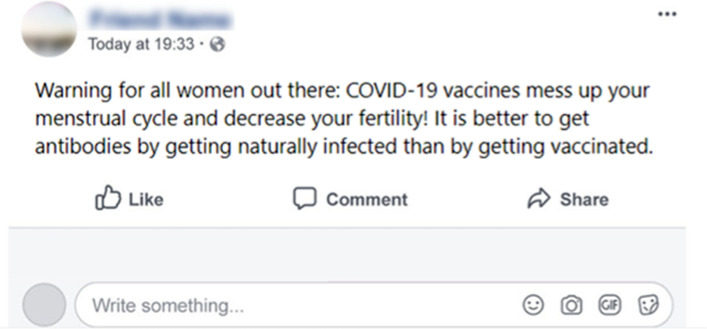
The Facebook post containing the vaccination misinformation.

We used a combination of common vaccination misinformation embedded in a (mock) social media post (Facebook) as the to-be-debunked misinformation. The post read: “Warning for all women out there: COVID-19 vaccines mess up your menstrual cycle and decrease your fertility! It is better to get antibodies by getting naturally infected than by getting vaccinated.” (Fig. 5).

As our debunking messages are intended for women of childbearing age, we addressed common vaccination-related misinformation in corrective texts, such as its impact on women’s fertility, safety during pregnancy, and the comparison between natural immunity and vaccination. Four different corrective texts and one control text were included in this study, representing the independent variables of text genre and harm presence: (1) Expository without appeal to harm (EnoH): An informative, didactic text that focused on scientific studies and statistics disproving the misinformation and providing accurate scientific information; (2) Expository with appeal to harm (EH): Text 1 but with an additional focus on harmful consequences; (3) Narrative without appeal to harm (NnoH): A narrative, anecdotal text telling the story of a pregnant, unvaccinated woman encountering complications during pregnancy/childbirth; (4) Narrative with appeal to harm (NH): Text 3 but with added harm (the mother died from COVID-19 complications); (5) Control condition: a text described skin and nail care, which was irrelevant to COVID-19. All texts were checked for scientific accuracy by vaccinologists on the topic. Based on the Flesch Reading Ease test, all four targeted texts were equally readable.

### Measures

#### Manipulation check

A manipulation check was conducted to ensure the validity of the experimental design. Participants were asked to rate their agreement with the statement (i.e., “The text talks about the harmful consequences of not getting COVID-19 vaccines”) on a 5-point Likert scale. Participants exposed to corrective texts with harm-stressing messages (*M* = 3.73, *SD* = 1.08) rated significantly higher than those exposed to messages without harm-stressing messages (*M* = 3.30, *SD* = 1.33), *t*(333.82) = -3.31,* p* = 0.001.

#### Vaccine skepticism

Vaccine skepticism was measured by including two items from *Belief in COVID-19 conspiracy theories*^85^ (i.e., “The so-called COVID-19 pandemic is nothing more than a smokescreen for covert actions of powerful forces” and “It is clear that the pharmaceutical industry is behind this pandemic”) and 7 items from *Vaccine Conspiracy Belief Scale*^86^ (i.e., “Vaccine safety data is often fabricated”, “Immunizing children is harmful and this fact is covered up”, “Pharmaceutical companies cover up the dangers of vaccines”, “People are deceived about vaccine efficacy”, “Vaccine efficacy data is often fabricated”, “People are deceived about vaccine safety”, “The government is trying to cover up the link between vaccines and autism”). Participants indicated how much they agree or disagree with the given statement on a 7-point Likert scale ranging from ‘strongly disagree’ (1) to ‘strongly agree’ (7). In terms of vaccine skepticism, individuals with an average score higher than 3.5 were categorized into the vaccine skeptic (SKEP) group (*n* = 155), while those with an average score lower than or equal to 3.5 were considered as in the non-skeptic (NONSKEP) group (*n* = 189).

#### Emotional response

A smiley-face assessment scale^87^, for measuring affective reaction (valence) with five faces ranging from very unpleasant (1) to very pleasant (5) (*M* = 3.50, *SD* = 1.19).

#### Debunking efficacy

Debunking efficacy was calculated as the difference between participants’ belief in vaccination misinformation after and before exposure to corrective information. Previous research indicates that using the same statement in both pre- and post-measures can lead participants to recognize the experimental manipulation, potentially introducing response biases^88^. Therefore, we used different but conceptually consistent measures for the pre- and post-measures, both focused on vaccine-related reproductive health misinformation. Before exposure to the correction, participants were presented with a mock social media post (Fig. 5) and asked, “To what extent do you believe the post you read?” from ‘completely false’ (1) to completely true’ (7) on a 7-point scale. Following exposure to corrective information, participants rated their agreement with three statements related to vaccine misinformation (i.e., “Vaccines reduce sperm quality in men”, “Vaccines disrupt women’s menstrual cycle length” and “Vaccines endanger the unborn baby during pregnancy”) on a 7-point scale, from strongly disagree (1) to strongly agree (7). The average score was then reversed. Thus, greater debunking efficacy was indicated by higher scores, which corresponded to a larger reduction in belief in misinformation after exposure to correction (*M* = 0.29, *SD* = 1.46).

#### Social media engagement

A social media engagement score was computed by averaging two items, including liking (i.e., “How likely are you to give this post a “like” if you would encounter it on social media?”), sharing (i.e., “How likely are you to share this post with your friends or followers on social media?”) on a 5-point Likert scale (*α* = 0.86, *M* = 3.23, *SD* = 1.32).

#### Information seeking intention

Participants indicated “How likely are you to look for more information about COVID-19 vaccines after reading the text?” on a 5-point Likert scale (*M* = 3.67, *SD* = 1.20).

#### Intrinsic barriers to COVID-19 information

Intrinsic COVID-19 health information seeking barriers were assessed by averaging four items (i.e., “I took a lot of effort to get the information I needed”, “I felt frustrated during my search for the information”, “I was concerned about the quality of the information”, and “The information I found was hard to understand”)^69^. Participants were asked to indicate the extent to which they had searched for information about the coronavirus and/or COVID-19 vaccines over the past two years, using a 5-point Likert scale ranging from ‘strongly disagree’ (1) to ‘strongly agree’ (5) (*α* = 0.66, *M* = 3.42, *SD* = 0.83).

#### Pregnancy complications

Participants were asked, “Have you experienced any complications during or after pregnancy?” (Yes or No), to capture their reported experiences with pregnancy-related complications.

### Procedure

We adopted a between-subjects study design, meaning each participant was randomly assigned to one of the five conditions (EnoH, EH, NnoH, NH, Control). First, we informed participants that we are interested in factors influencing attitudes toward COVID-19 vaccination. No time limit was imposed, allowing them to pay close attention and take the time they need. Participants were initially asked to provide informed consent. After expressing their voluntary agreement to participate in the study, they were presented with the misinformation in a Facebook post format (Fig. 5). Immediately after the exposure to misinformation, participants were asked a set of questions about it (i.e., emotional responses, beliefs, social media engagement, see measures). Participants were then randomly assigned to one of the five texts. After reading a text, we used the same set of measures that were presented after the misinformation to investigate participants’ emotional responses, beliefs and social media engagement towards the corrective text. In order to ensure that all participants exposed to misinformation have access to the debriefing information, we provided debriefing materials at the end of the survey. These materials comprised a text with verified facts and hyperlinks to authoritative and credible sources (e.g., WHO, CDC, and Vox)^89^.

## Data Availability

The data, scripts, and supplementary materials can be found on our Open Science Foundation (OSF) page: https://osf.io/j6vgy/?view_only = ad8a3cf26db24abc92e3661aa33afcd4.

## Appendix

See Tables 4 and 5.

**Table 4:** Descriptive statistics for debunking efficacy for corrective and non-corrective control text conditions.

Condition	*N*	*M*	*SD*	*Min*	*Max*
Expository with harm (EH) corrective	81	0.51	1.46	− 4.00	5.00
Expository without harm (EnoH) corrective	91	0.38	1.44	− 3.33	4.17
Narrative with harm (NH) corrective	87	− 0.00	1.45	− 4.00	5.00
Narrative without harm (NnoH) corrective	85	0.27	1.48	− 3.33	6.00
Non-corrective control	88	− 0.53	1.17	− 4.00	2.00

**Table 5:** Post hoc mean comparison results of debunking efficacy (corrective vs. non-corrective control text conditions) with Bonferroni correction.

Corrective vs. Non-corrective control condition	*MD*	*Sig*	95% Confidence Interval	
			Lower Bound	Upper Bound
EH corrective vs. Non-corrective control	1.04	< 0.001	0.43	1.65
EnoH corrective vs. Non-corrective control	0.92	< 0.001	0.33	1.51
NH corrective vs. Non-corrective control	0.53	0.13	-0.07	1.13
NnoH corrective vs. Non-corrective control	0.80	0.002	0.20	1.40

EH = expository with harm, EnoH = expository without harm, NH = narrative with harm, NnoH = Narrative without harm. *MD*: Mean Difference.
